# Towards research-based learning outcomes for general practice in medical schools: Inaugural Barbara Starfield Memorial Lecture

**DOI:** 10.3399/bjgpopen17X100569

**Published:** 2017-01-09

**Authors:** Denis Pereira Gray

**Affiliations:** Past Chair of Council and President, RCGP; Emeritus Professor; Consultant, St Leonard’s Research Practice, Exeter, UK

**Keywords:** general practice, primary care, learning outcomes, curriculum, undergraduate, medical schools

‘… Medical schools should recognise they have a responsibility to patients to educate and prepare half of all graduates for careers in general practice … Teaching and promotion of general practice as a career which is as professionally and ***intellectually*** rewarding as any other specialism. Those medical schools which do not teach primary care ***as a subject*** should be held to account by the General Medical Council.’^1^

‘… It [*general practice*] is a really hard job. They GPs, have to be clinically, ***intellectually*,** and emotionally strong … to identify major clinical problems masquerading as minor ailments and it is utterly relentless. It requires a lot of***intellectual*** flexibility and people have to be very tolerant individuals. It is one of the hardest jobs in medicine.’^2^

‘… Health Education England is currently working with the Medical Schools Council, higher education institutions, the RCGP and the GPC to increase the profile of general practice in medical schools and in ***their curricula***. A working group, chaired by Professor Valerie Wass OBE, will publish recommendations in Summer 2016 about recruitment and selection, finance and ***curriculum***and the promotion of general practice ***as a specialty*.**’^3 ^(author’s italics)

## Studying the discipline of general practice

These statements underline why medical students should study general practice as a specialty or specialism, that is as a distinct subject, and that this needs to be done intellectually, a word appearing three times in these extracts. ‘Intellectual’ is defined by the *Oxford Dictionary* as ‘possessing a high level of understanding …’ implying understanding of general practice research.

An intellectual approach is needed as current medical students have been highly selected on intellectual criteria. They want know the ‘hows and whys’ of medicine. If they hear theory and principles only from hospital specialists, they will be drawn only to hospital practice and 81% of medical students currently do not see general practice as their first choice.^4^

The words ‘primary care’ are not used here, except in quotation, and ‘general practice’ is used. The meaning of primary care is unclear. It mixes community and hospital services, relationship and non-relationship-based services, and five different health professions. This curriculum is designed to help medical students understand and choose general practice, enthusiastically. The avoidance by medical schools of the words ‘general practice’ in their prospectuses and within their institutions is prejudicial, making it harder for medical students to choose general practice positively as a career and be proud of it.

## Learning in medical schools

Despite calls for a UK core curriculum in general practice,^5,6^ none currently exists. There is confusion between general practice as a place for learning and as a discipline to be learned.

### All students

Medical schools prepare students for all branches of medicine. The GP curriculum should be provided within the medical school curriculum to all students, including those seeking hospital careers, so that all future doctors understand the theory and principles of general practice. The medical school’s course, of at least 20 hours, should be integrated with teaching in the general practices, so that local GPs, well-briefed on the GP curriculum and with practice libraries and files of key articles, can demonstrate the integration of theory and practice. Some medical schools provide 20% of the undergraduate curriculum time in general practice,^7^ so 20% of the teaching resources of the medical school should follow.

### Learning about general practice as an independent discipline

The theory and principles of general practice should not be confused with students learning about the diseases seen in general practice. The diseases are usually learned well, whereas general *practice as a distinct subject* is usually not learned at all.

### Teaching a subject or university discipline

A discipline requires a defined body of knowledge^8^ and for universities and medical schools, this knowledge is published in peer-reviewed journals.^9^

A modern curriculum must source its statements, so learners and teachers are linked repeatedly with relevant research: research-based teaching is fundamental.

The reading list of the Tamar Faculty of the Royal College of General Practitioners could be built on.^10 ^This is the first set of referenced, that is research-based, learning outcomes collectively forming a GP curriculum, which should be delivered within the medical school’s overall curriculum. While incomplete, it offers a start in fostering development.

### Teaching and assessment within the medical school

The GP curriculum should be taught within the medical school, not outsourced. If in 5 years of higher education, medical students see nothing positive about general practice in the medical school’s prospectus, see no GP curriculum, no clear learning outcomes, no exciting GP reading-list, then the medical school is:

‘… giving a powerful non-verbal signal that general practice is not important and that nothing has been written from or about general practice that future doctors need to read!’^1^

It is essential that examination and assessment systems in all UK medical schools reflect the importance of general practice, so 20% of examination questions should be on the theory and principles of general practice.

## Theory and principles of general practice: learning outcomes

### Facts about general practice

*Students need to understand the scale and importance of general practice. *Students need to know the ‘dozen facts about general practice’,^11^ which is the biggest and most important branch of the medical profession, managing 86% of the problems that the population brings to doctors and nurses^12^ on only 8.5% of the NHS budget.^1^ Its UK-wide Medical Royal College, has the biggest membership and the biggest annual income of the all the Medical Royal Colleges in the British Isles.

### First-contact care

*General practice is the front line of the NHS.* ^13 ^Students should understand that GPs see patients with undifferentiated illness in any part of the body or mind. A big challenge is ‘major illness masquerading as minor illness.’^2^ Presenting symptoms in general practice can be physical, psychological, or both and are heavily influenced by the social determinants of illness,^14^ which GPs see first-hand in individual patients more than any other doctor.

Culture affects what is perceived as illness, how illness presents, and why some treatments may cause difficulties for some cultural groups. Helman, a GP Professor of Anthropology, wrote classic books on culture and health.^15,16^

### Diagnoses

*Medical students should understand that general practice is where most diagnoses are made*. Some diagnoses are never or rarely made in hospitals. Students should understand the importance of making diagnoses, their value for treatment and prognosis, and the reassurance diagnosis gives patients. Students should also understand the adverse-effects of diagnoses, including labelling and stigma for some patients.

Diagnoses in general practice are made under time pressure and with fewer investigations available. Errors occur. One study found 9.4% of primary care diagnoses were wrong.^17^ Students should learn how to reduce this.

### Human behaviour

*Students need to learn to think about patients in terms of behaviour as well as pathology*. One of the main reasons why general practice differs from hospital practice is the importance of human behaviour by patients, families, and doctors. Feelings loom large.

Research on the behaviour of patients before the consultation^18,19^ during the consultation^20,21^ and compliance after the consultation^22^ needs to be understood. How do doctors build a ‘sustained partnership with patients’^23^ and achieve patient-centred consulting?^24^ How do such medical behaviours affect medical outcomes?^25^ Many difficult diagnoses, like pathologically-unexplained symptoms, and many management problems in general practice are behavioural.

GP thinking first conceptualised human behaviour as infectious,^26^ which has been supported by dramatic recent research.^27,28^

### Medical generalism

*Mens sana in corpore sano (a sound mind in a sound body). *Both the ancient Greeks and the Romans understood that body and mind are closely interrelated, as has been confirmed by recent research.^29^

GPs are unique in being the only doctors who accept both problems of the mind and the body simultaneously. For over 200 years, they have successfully resisted the separation of body and mind, often into separate hospitals, which is the great flaw in specialist medicine, worldwide. Medical generalists take the widest view of the patient and alone can see their patients and their illnesses in context.^30^ It is a great advantage for patients to feel understood and to have, say, depression and diabetes, diagnosed and treated in a single consultation.

Students should understand the comparative advantages and disadvantages of generalist and specialist care. This should include research showing better outcomes by specialists than generalists,^31^ research where NHS GPs matched consultant psychiatrists for depression^32^ and US family physicians matched orthopaedic specialists for backache,^33^ and research where GPs outperformed specialist mental health services in detecting suicide risk.^34^

### Continuity of care

*Continuity is the ‘mortar that holds general practice care together’ (Bradley, NCA [2015]); personal communication)*. Medical students should understand research showing that continuity of care benefits patients through better quality care,^35^ and reducing emergency hospital admissions^36^ and even mortality.^37^ Doctors benefit by working with more satisfied,^38^ trusting,^39^ and compliant, patients.^40^

Continuity benefits society through better uptake of preventive medicine,^41^ fewer hospital admissions,^36^ and lower-cost health systems.^42,43^ Students should understand the adverse effects of continuity, like dependency.^44^

### Family care

*The commonest name for GPs worldwide is ‘family physician’*. Many illnesses are familial: alcoholism, several common cancers (including breast and prostate), depression, diabetes, epilepsy, glaucoma, ischaemic heart disease, obesity, several rheumatic conditions, and schizophrenia. Family doctors knowing the family history can diagnose more quickly and are better at risk assessment. Most couples with children register with the same general practice, so students should understand this unique potential for medical care. Huygen’s classic *Family Medicine* stated that ‘the family is the unit of care.’^45^ Students should understand the privilege of family doctors having several family members as patients simultaneously and caring across generations.

Students should appreciate the arithmetic of English GP consultation rates. The average patient contacts their general practice 5.2 times per annum, seeing a GP face-to-face three times a year.^46^ Therefore, a four-person household will see a GP on average 12 times a year. Assuming 10-minute consultations, this means 2 hours per annum. If GPs group families on personal lists,^47^ they have plenty of time to get to know their patients as people. Otherwise, consultations are dispersed between different doctors.

When I retired from clinical practice, 7% of my personal list (averaging one patient in every surgery session) had a four*-*generational relationship with me: a baby, one or more parent, one or more grandparents, and one or more great grandparents registered simultaneously. It’s a rich experience being a real family doctor.

### Personal preventive care

*Preventing illness is the doctor’s ‘supreme objective’.* ^26 ^General practice uniquely integrates preventive medicine with curative medicine. Students should appreciate how much primary preventive care GPs provide to registered populations and symptom-free people.

Secondary prevention includes detecting conditions before symptoms arise; for example, abnormal cervical smears and hypertension.

GPs are the experts in identifying and minimising risk factors for disease; for example, detecting and treating high blood pressure and raised cholesterol for millions. Such clinical opportunistic screening, a cost-effective process, has helped to halve UK heart attacks in the last decade.^48^

Students should understand that tertiary prevention, the prevention of complications of existing disease, is a central part of GP work with computerised risk assessment for cardiovascular disease routine. General practice more than any other branch of medicine provides the greatest opportunities for health promotion.

### Care in the patient’s home

*Homes influence the health and happiness of those living there. *One model of the consultation is the GP should understand: ‘what the patient is feeling at home.’^26^ Working comfortably in patients’ homes is a skill to be learned and how homes influence the origin of disease and the ability of people to manage illness there. Patients disclose information more freely at home and rapport points can facilitate care.^49^

It is usually only in homes that GPs see family groups together; for example, seeing a grandmother or lodger, influencing family dynamics. For disabled people and the dying, home visiting is especially important.

### Care in the community

*GPs are the lead community clinicians with unrivalled opportunities to collaborate with other community-based services and voluntary groups. *While hospitals are distant from many homes, only 1% of English city dwellers live more than 2 kilometres away from a general practice,^1^ so most practices are within ‘pram-pushing’ distance.

### Patient-centred medicine

*In the medical profession general practice is best placed to achieve patient-centred care. *The aim in medicine is to understand and treat each patient as a person. Generalist doctors can best integrate the widest range of medical conditions, the patient’s psychological state, and often relevant social factors. Getting to know patients as people is much easier in general practice.

Doctors should understand each patient’s ‘concerns, ideas, and expectations.’^50^ To provide high-quality personal care the doctor must understand the patient’s hopes and fears and how illness affects them. It requires skill to gather this information and repeated consultations with the same patient are how valuable ‘accumulated knowledge’^51^ is gained.

### Patient–doctor relationships

*General practice has the longest and deepest working relationships with patients*. Students should understand that good patient–doctor relationships are particularly important in general practice and that not all doctors are suitable for general practice.^20^

GPs must be tolerant and flexible to meet the needs of their patients. GPs alone define their medical role through relationships with patients.^30^ Students should reflect on how such relationships are created and maintained. After 20 consultations with the same doctor, about 80% of patients consider their relationship with their family doctor is ‘deep’.^52^

Students should know the benefits of strong patient–doctor relationships and their adverse-effects, like dependence. Students should understand contextual healing (placebo effect)^53^ and how it can extend effectiveness in general practice.

#### Empathy

Doctors in histopathology, accident and emergency medicine, and urgent-care centres, have chosen relatively relationship-free settings. But, empathy matters in general practice, which is different.^54^ Medical students should know the systematic review indicating that their empathy levels on average decline as they progress through medical school^55^ and what can be done about it.^56^

Students should know that general practice provides the longest and richest patient–doctor relationships in medicine. The median duration of registration of the 8800 patients in my former practice in 2016 is 7.1 years and the upper quartile of patients have been registered for 18.9 years.^57^

#### Biographical approach

General practice fosters the biographical approach to medicine, such as understanding significant life-events^58^ and the adverse effects of childhood abuse.^29^ These enable GPs to understand both the antecedents of much ill health and their patients as people.

#### Narrative medicine

*Patients mainly communicate verbally with doctors through particular words and metaphors. *While all doctors use narrative medicine, generalists have studied it most,^59–61^ and the widest applications are in general practice.

### Cost-effectiveness

*Medical students should understand why general practice is exceptionally cost effective. *Primary care orientated health systems have lower overall costs.^42^ Healthcare costs are influenced by the values of sites, buildings, and equipment. Hospital services through their overheads are more expensive than general practice. Students should understand how GPs manage 86%^62^ of the medical needs of the population on (in 2013–2014) only 8.2%^63^ of the English health budget.

A single outpatient appointment with an English Consultant Diabetologist cost £222 in 2015.^63^ The cost of a GP appointment in 2015 was £37,^64^ an appointment in a nurse-led clinic was £39 in 2010–2011,^65^ and an ambulance attending a home in 2015 cost £231.^66^ Students should understand why general practice takes pride in its cost efficiency.

### Population medicine — the registered list

*General practice alone has a precisely-known population with characteristics like age, sex, and social deprivation available. *A British GP was the first doctor in the world to go online with a medical record.^67^ For the most IT literate generation of medical students, GP computing, the most sophisticated in the English NHS, offers rich opportunities for quality assurance, education, and research.

### Practice management

*Most GPs are independent contractors, uncommon in the NHS, but the norm for community professionals*.^68 ^Partnership working is a good model for partnership working with patients. Partnership is a privilege, giving locus of control^69^ and flexibility which, with the absence of hierarchies means that half of female GPs have their first baby by age 32 years, whereas less than half the female doctors in anaesthetics, general medicine, and surgical specialties, have their first baby by age 35 years.^70^ This 3-year difference is highly significant for women in their 30s. Salaried doctors are in a ‘master–servant’ relationship^71^ hence GP partners, unlike salaried hospital consultants, do not have gagging clauses in their contracts.

### Patient satisfaction

General practice patients are more satisfied with ‘Your GP’ than hospital inpatients or outpatients.^72^ They are also far more satisfied than with consultations in accident and emergency departments.^73^

### Reducing socioeconomic disadvantage

*Inequality and health inequalities are one of the biggest challenges of our time. *Social deprivation is associated with higher rates of illness and deaths. The mortality rate for men in routine occupations is almost three times higher than men in professional work.^74^ Students should know why hospitals are ‘pro rich’ and general practice is ‘equitable.’^75^ and how GPs, more than other clinicians, can ameliorate social disadvantage.^76–78^

Medical students and junior doctors have strong social consciences and many entered medicine to help people. General practice is the clinical setting where more can be done for more people than anywhere else.

### Reducing mortality

*Death is the ‘hardest’ of all outcomes. *‘Each additional family physician is associated with 34 lives saved per 100 000 citizens.’^79^ More lives are saved in general practice than by any other branch of medicine.

### GP education

General practice is quietly taking the lead in UK medical education. As early as the 1970s, general practice, alone, required GP trainers to be trained as teachers and dismissed trainers if they underperformed.^80^ Junior doctors reporting on their training, rank general practice highest.^81^ There is an unacceptable amount of bullying of junior doctors in specialist training.^82^ The two branches of medicine where the patient’s feelings are central on the doctor’s professional agenda, general practice and psychiatry, had the least bullying. General practice was best of all. Thirdly, the General Medical Council survey of work-based placements in medicine, found those in general practice were ranked highest.^83^

However, a collective blind spot in reading research in both undergraduate and postgraduate GP education exists. Only when this is rectified, will GP education consolidate its leading position.

## Career opportunities

Most children never see a paediatrician after newborn checks. Most older people never see a geriatrician. Most mentally ill patients never see a psychiatrist, and most people with diabetes never see a diabetologist. Yet GPs see all of these patients, every week.

General practice has at last been recognised as the central discipline in medicine. It is now the top priority for medical careers in the UK, with half of all postgraduate training places reserved for it.^1^

## Conclusion

The recommendation of the UK Parliament^1^ in April 2016 that primary care and general practice be taught in UK medical schools ‘as intellectually rewarding’ and ‘as a subject’ was historic and the most important event in undergraduate general practice teaching since 1948. The second step is agreeing what is to be taught in UK medical schools, hence this research-based curriculum. The third remaining step will be assessing these learning outcomes.

## Addendum

Since this article was accepted for publication, the report of the Wass Committee has been published.

Wass Committee (2016) *By Choice not by Chance Supporting medical students towards future careers in general practice*. Chair, Professor Valerie Wass. London: Medical Schools Council and Health Education England.

It includes a paragraph:

“Formal teaching on the core knowledge and skills **which distinguish general practice as a specialty** distinct from those of broader generalist practice is *needed*.” P28. [Author's emphasis].
